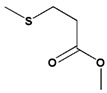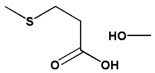# Recent Advances in pH- or/and Photo-Responsive Nanovehicles

**DOI:** 10.3390/pharmaceutics13050725

**Published:** 2021-05-14

**Authors:** Yuseon Shin, Patihul Husni, Kioh Kang, Dayoon Lee, Sehwa Lee, Eunseong Lee, Yuseok Youn, Kyungtaek Oh

**Affiliations:** 1 Department of Global Innovative Drugs, The Graduate School of Chung-Ang University and College of Pharmacy, Chung-Ang University, 221 Heukseok-dong, Seoul 06974, Korea; sus9417@cau.ac.kr (Y.S.); patihul.husni@unpad.ac.id (P.H.); rldh4122@cau.ac.kr (K.K.); dayoon1231@cau.ac.kr (D.L.); summermaria@cau.ac.kr (S.L.); 2 Division of Biotechnology, The Catholic University of Korea, Bucheon 14662, Korea; eslee@catholic.ac.kr; 3 School of Pharmacy, Sungkyunkwan University, Suwon 16419, Korea; ysyoun@skku.edu

**Keywords:** chemotherapy, nanovehicles, light, pH, stimuli, photothermal, photodynamic, protonation, acid-labile bond

## Abstract

The combination of nanotechnology and chemotherapy has resulted in more effective drug design via the development of nanomaterial-based drug delivery systems (DDSs) for tumor targeting. Stimulus-responsive DDSs in response to internal or external signals can offer precisely controlled delivery of preloaded therapeutics. Among the various DDSs, the photo-triggered system improves the efficacy and safety of treatment through spatiotemporal manipulation of light. Additionally, pH-induced delivery is one of the most widely studied strategies for targeting the acidic micro-environment of solid tumors. Accordingly, in this review, we discuss representative strategies for designing DDSs using light as an exogenous signal or pH as an endogenous trigger.

## 1. Introduction

Successfully developed cytotoxic drugs have enabled advances in chemotherapy, which has greatly improved the prognosis and quality of life of cancer patients [1]. Chemotherapeutic agents in combination with nanotechnology have resulted in more effective drug design and development of cancer treatments [2,3,4]. Various materials have been developed for nano-sized drug delivery systems (DDSs), which are capable of targeting tumor sites spatiotemporally for the desired treatment [5]. Nanovehicles physically or chemically incorporated with drugs effectively deliver the payload to solid tumors through long systemic circulation without extravasation and passive targeting, enhanced permeability and retention (EPR) effect [6,7,8]. The favorable properties of nanomaterials have led to impressive progress in the development of innovative nanovehicles for therapeutic agents, which have shown enhanced efficacy and minimized toxic side effects of the incorporated anticancer agents due to increased accumulation of drugs in the target tissues [9,10,11,12,13,14,15]. However, the nanovehicle itself may often be trapped in the cellular endosome, or the nanovehicle as a protective barrier may not be completely disrupted at the tumor site; thus the drugs in the nanovehicles may not be released efficiently [16]. Consequently, a stimuli-responsive delivery system exhibiting controlled release of therapeutic payloads in response to a given stimulus can serve as a promising strategy for enabling precision delivery of drugs and improving antitumor activity [17]. Stimulus-sensitive DDSs can be designed to respond to stimuli (both alone and in combination) in the endogenous environment (pH, enzyme activity, redox reactions, and others [18,19]) or exogenous or externally applied triggers (e.g., light, temperature, ultrasound [20]). Responsiveness to a specific internal or external stimulus in the diseased site could enhance the precisely controlled release and accumulation of preloaded therapeutic agents from nanovehicles delivered to target sites, leading to higher efficiency of antitumor treatment [21,22,23].

**Scheme 1:**
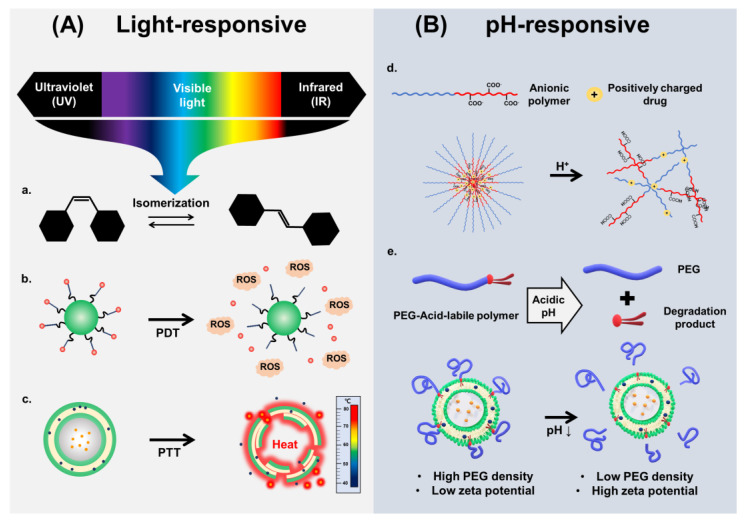
Schematic illustration of the concept of (**A**) light- or (**B**) pH-responsive drug delivery systems (DDSs); **a.** photo-isomerization, **b.** photodynamic responsive DDSs, **c.** photothermal-responsive DDSs, **d.** protonation-based DDSs, **e.** acid-labile bond cleavage-based DDSs.

Among previously mentioned methods for external stimulation, light is a particularly attractive strategy for therapeutic applications owing to easy adjustment of its intensity, non-invasive application, and exquisite temporal and spatial control. Light-responsive systems that absorb light, a type of electromagnetic wave, use light energy to trigger changes in the chemical bonds, polarity, and chemical groups or induce the generation of heat and reactive oxygen species (ROS) (Scheme 1A). On this basis, photo-responsive DDSs have been widely explored for enabling the release of tumor-targeting drugs at a therapeutic index by precisely controlling the light irradiation site, dosage, and time. Light-based innovative delivery platforms generally use two patterns: photo-induced chemical transformation (Scheme 1a) and photo-mediated intermediate reaction (Scheme 1b,c). Photochemical platforms can be transformed when the chemical structure of the material absorbs light at specific wavelengths. Comparatively, photo-mediated platforms, such as photothermal or photodynamic delivery systems, can generate heat or ROS under light irradiation, triggering the transformation of nanovehicles to promote drug release.

**Scheme 2:**
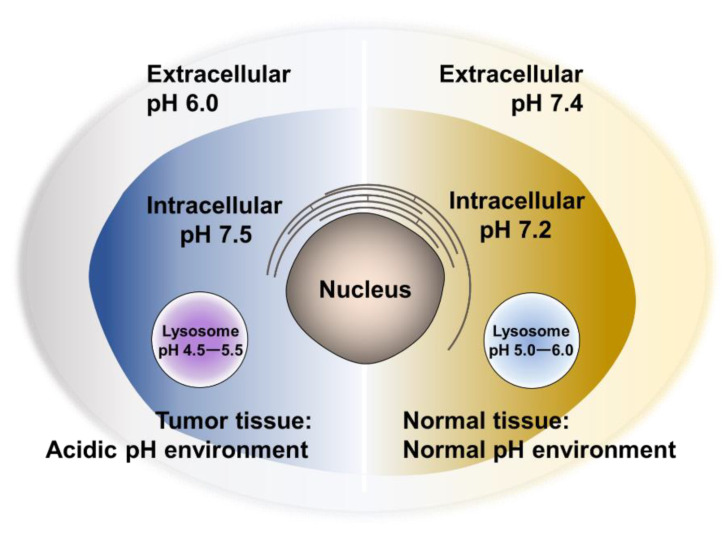
Schematic illustration of differences in pH at the cellular level.

pH-responsive systems have been most widely studied for designing nanosystems for anticancer drug delivery (Scheme 1B). As shown in Scheme 2, most solid tumors have a lower extracellular pH (pH_ex_) than normal tissues, with a mean value of 6.8 (ranging from 5.7 to 7.8) [24]. In general, cancer cells use glucose for glycolytic metabolism and produce lactic acid faster, regardless of hypoxia, than normal cells to acquire the energy required for their survival; the high metabolic rate of these tumor cells has been studied as a major cause of the acidic tumor micro-environment (pH ranging from 6.5 to 7.2) [25]. In addition, intracellular endosomes and lysosomes have a considerably lower pH of 4.5–6.5 (endosomal pH (pH_en_)) [26,27]. pH-responsive systems have been developed using chemical structural changes, such as changes in hydrophilicity by deprotonation and protonation (de/protonation) and degradation of chemical bonds by acid-catalyzed cleavage. These pH-responsive systems can be used for preventing various drugs and carriers for cancer treatment from being trapped in endosomes [28,29]. Therefore, pH-responsive DDSs are important for controlling drug delivery in tumor diseases.

In this review, recently developed photo-responsive nanovehicles and pH-responsive nanovehicles are discussed, with a focus on the representative strategies for designing pH- or photo-responsive nanovehicles. Additionally, we address pH- and photo-dual stimuli-responsive nanovehicles for maximizing antitumor activity. This review mainly focuses on the principles and benefits of these nanovehicles, stimuli-responsive polymers, and various critical chemical bonds and functional groups of the materials that are exploited to achieve the pH- and/or photo-responsiveness of DDSs.

## 2. Photo-Responsive Nanovehicles

Light-responsive nanovehicles using diverse light sources, such as ultraviolet (UV), visible, and near-infrared (NIR) light, exhibit more controllable drug release through the spatiotemporal control of light [30]. The reaction process in a nanovehicle can be controlled by the light intensity, emission wavelength, pulse length, and exposure time [31]. The photo-responsiveness indicates two patterns of light-induced chemical transformation and a light-generated intermediate reaction. In the first category, the chemical structure, including photo-responsive molecules, can be transformed through reactions such as photo-isomerization and photo-cleavage upon absorption of light with specific wavelengths. Comparatively, the second group of photo-responsive nanovehicles generates intermediate molecules via photosensitive agents (PSAs) such as metal nanoparticles, carbon nanotubes, and organic dyes. Photo-responsive nanovehicles have been applied for optical imaging, phototherapy, and theragnosis for preventing and treating tumors. Table 1 is a summary of photo-responsive nanovehicles.

### 2.1. Photo-Responsive Nanovehicles Using Photo-Induced Chemical Transformation

**Figure 1:**
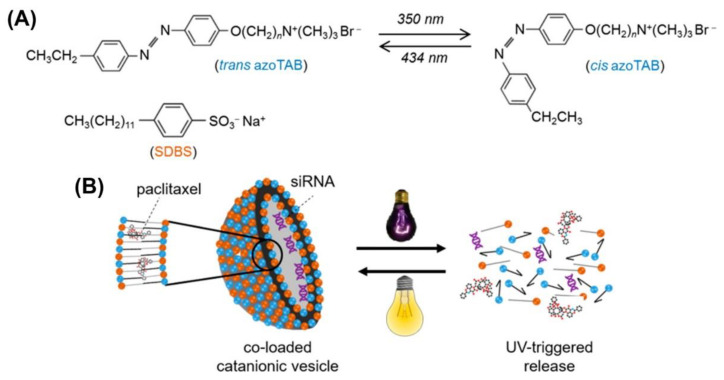
(**A**) Structure of azoTAB and SDBS surfactants, as well as the trans to cis photo-isomerization of azoTAB upon exposure to visible (434 nm) or UV (350 nm) light. The lengths of the alkyl spacers in azoTAB analogs are *n* = 2 (2-azo-2), *n* = 4 (2-azo-4), or *n* = 6 (2-azo-6). (**B**) Illustration of the reversible transition of cationic vesicles to free surfactants with UV illumination. Reproduced from [32] with permission, copyright 2020, American Chemical Society.

Photo-responsive nanovehicles using photosensitive chemistry have been developed and studied, providing the capacity for incorporation of drugs, targeting tumor sites, controlled drug release through direct modification of the chemical structure of materials, or decomposing the materials under light illumination. First, cis and trans isomerization of photo-reactive nanovehicles is a common strategy that has been used for changing the polarity of materials with azobenzene or spiropyran (SP) and for promoting the release of the drug payload [47]. In the case of azobenzene, when materials include a conjugated π system with strong absorption from UV to visible red light, the cis isomer of azobenzene is converted to trans-azobenzene [48]. It shows a strong π−π transition in the UV region and a weak π−π transition in the visible region. Photo-isomerization of the azobenzene group can regulate light-induced drug release. For instance, Seidal et al. developed an azobenzene-based photo-responsive carrier composed of azobenzene trimethylammonium bromide (azoTAB) and sodium dodecylbenzenesulfonate (SDBS), which showed reversible photo-isomerization for the treatment of breast cancer. As shown in Figure 1, the vesicle carriers formed from the longest trans-azoTAB showed higher siRNA and paclitaxel encapsulation and more effective transfection. The drug co-loading vesicle carriers can enhance cell death and anti-apoptotic B-cell lymphoma-2 protein suppression due to UV-triggered release of the drugs from the ruptured vehicles [32].

SP has additionally been studied as an isomerization species; it reversibly transforms into different structures through photoreaction. SP refers to a closed cyclic isomer that exhibits hydrophobicity; it becomes another form of merocyanine (MC) under UV irradiation. In SP, which has a non-planar form because of the vertical position of indolenine and benzopyran, the photoreaction cleaves the spiro C–O bond, yielding a hydrophilic plane state of the MC form [49]. In contrast, in the MC type, it absorbs visible light, which triggers SP-type isomerization [50,51]. Reversible structural isomerization physically and chemically affects the connected substances and surrounding structures. Among recent studies, Bahareh Razavi et al. reported multi-responsive micellar assemblies composed of poly(dimethylaminoethyl methacrylate) (PDMAEMA) and poly(methyl methacrylate) (PMMA). SP-(PDMAEMA-block-PMMA) and SP-(PMMA-block-PDMAEMA) were synthesized via atom transfer radical polymerization (ATRP) using an SP ATRP initiator. The doxorubicin (DOX)-loaded micelles prepared from the block copolymers increased DOX release in response to changes in temperature and pH, which significantly increased under UV irradiation. This was due to an increase in water solubility and a change in micelle morphology caused by the isomerization of SP to MC by UV irradiation [33].

**Figure 2:**
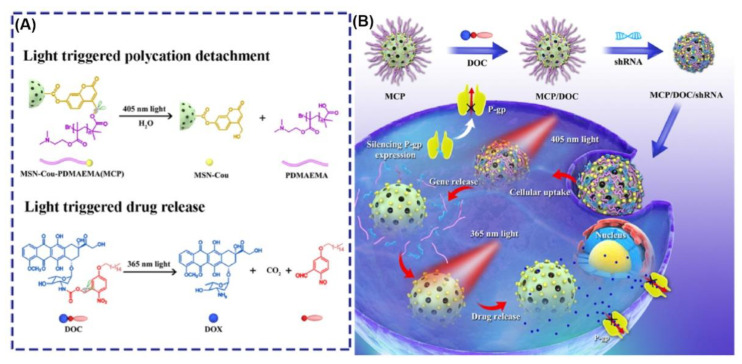
(**A**) Chemical structure and photolysis of photo-responsive vehicle (MCP) and DOX prodrug (DOC). (**B**) Schematic illustration of sequential release of shRNA and DOX regulated by 405 and 365 nm light irradiations, using photo-responsive mesoporous silica nanoparticles as co-delivery vehicles for optimizing the synergistic therapy in multidrug-resistant cancer cells. Reproduced from [34] with permission, copyright 2018, American Chemical Society.

Another nanovehicle strategy using UV irradiation is photo-induced cleavage using agents such as *o*-nitro benzyl and coumarinyl ester. The drugs connected through these linkages can be released through irreversible cleavage, under the light illumination of the appropriate wavelength. *o*-nitro benzyl and its derivatives cause a series of radicular mechanisms and hydrogen abstraction by UV light (365 nm) irradiation, releasing the connected molecules [52]. Another favorable photo-inducible cleavage material, coumarinyl ester, has exhibited strong fluorescence and light energy release [53]. The coumarinyl ester is irreversibly cleaved in response to UV light. Usually, coumarin is incorporated into a polymer through an ester bond for imparting hydrophobic properties. The ester bond is cleaved by an external light stimulus and the remaining polymer backbone frame forming a carboxylic acid structure leads to the collapse of the nanovehicle owing to its hydrophilic nature [54]. Wu et al. developed a photo-responsive mesoporous silica nanoparticle (MSN) using both *o*-nitro benzyl and coumarinyl ester as a co-delivery vehicle for P-glycoprotein short-hairpin RNA (shRNA) and DOX. MSN was linked to PDMAEMA by coumarin ester bonds (MSN-Cou-PDMAEMA) (MCP) for the photo-responsive release of shRNA and incorporated with DOX in combination with hexadecyl-*o*-nitrobenzyl. The shRNA and DOX were released when sequentially irradiated with UV light of 405 nm and 365 nm, respectively, resulting in synergistic effects in multidrug-resistant HepG2/ADR human liver cancer cells (Figure 2) [34].

**Figure 3:**
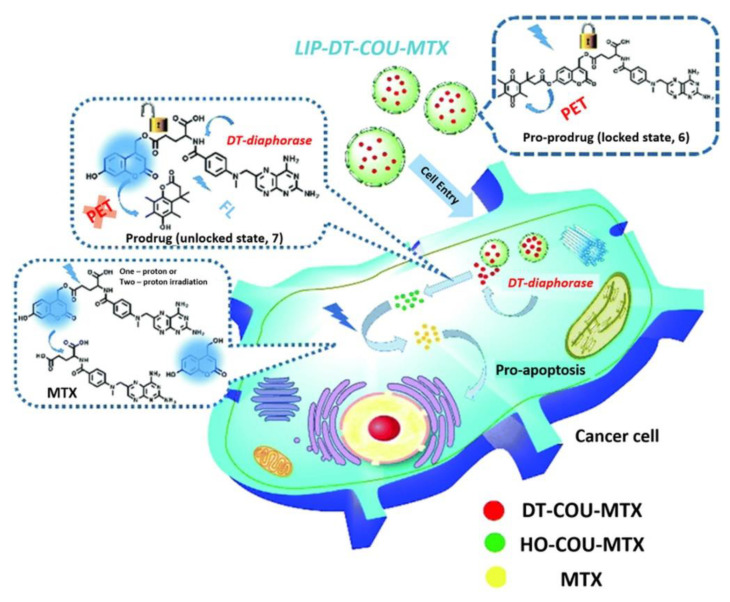
Schematic overview of a pro-prodrug nanosystem for imaging and therapy. Before enzyme activation, in the locked state pro-prodrug, the coumarin is a dormant photo-trigger with quenched fluorescence, while the enzymatic reaction leads to the cleavage of the quinone propionic acid group, which not only restores the fluorescence of coumarin but also makes it an active photo-trigger, leading to the release of the active drug. Reproduced from [35] with permission, copyright 2018, Royal Society of Chemistry.

Compared to UV light, which has limitations in clinical practice such as low penetration depth, risk of cell damage, and attenuation by blood and soft tissue, NIR rays with a wavelength of 700~1000 nm exhibit considerable penetration features and low toxicity to normal cells; the latter have been utilized as a favorable light source for triggering drug release in photo-responsive nanovehicles [55,56]. Wu et al. reported an enzyme-reactive, two-photon NIR-reactive pro-prodrug nanosystem for cancer detection and therapy. The nanosystem was composed of methotrexate (MTX), DT-diaphorase-responsive quinone propionic acid, and photo-responsive coumarinyl (DT-COU-MTX). DT-COU-MTX can release the drug when subjected to two external stimuli, enzyme and light. In the absence of DT-diaphorase, quinone propionic acid inhibited coumarin fluorescence and photo-responsive cleavage through photo-induced electron transfer. In contrast, in the vicinity of cancer cells overexpressing DT-diaphorase, the coumarin fluorescence of the pro-prodrug was activated and could be monitored to detect the tumor. Thereafter, MTX in the form of a prodrug (HO-COU-MTX) was released through cleavage of the bond by two-photon NIR irradiation, resulting in high cytotoxicity toward cancer cells with less effect on normal cells (Figure 3) [35]. Another NIR-reactive substance, diazo-1,2-naphthoquinone, undergoes Wolff rearrangement via photon induction. It is easily converted to the hydrophilic 3-indenecarboxylic acid (pK_a_ 4.5). This Wolff rearrangement reaction can be generated by one high-energy UV photon and two low-energy NIR photons. Therefore, it can be effectively applied to NIR-triggered drug release systems [57,58,59,60].

### 2.2. Photo-Responsive Nanovehicles Using Photo-Mediated Materials

Light can be converted into heat and generate ROS or gas from photo-responsive nanovehicles through mediators incorporated in the nanovehicle indirectly, along with chemical transformation or decomposition of the molecular structure in direct photo-responsive nanovehicles, as previously mentioned. Here, we briefly describe the utilization of mediators by light irradiation of photo-responsive nanovehicles using photothermal therapy (PTT) and photodynamic therapy (PDT).

#### 2.2.1. Photo-Responsive Nanovehicles Using PTT

The photothermal effect is most commonly used for indirect photosensitive drug delivery. Nanomaterials that absorb the light of a specific wavelength convert light energy into thermal energy, inducing drug release or hyperthermia conditions for PTT [61]. In addition, the photothermal effect can promote the extravasation of nanomaterials from the blood in the tumor area irradiated with light and can enhance intracellular absorption and drug release, thereby resulting in an improved therapeutic effect [62,63,64]. NIR-reactive nanostructures have been synthesized, and they are being actively studied as efficient photothermal nano-formulations for cancer PTT because NIR can penetrate deep tissues and show little toxicity [65,66,67]. Various mediator materials with the ability to convert NIR light into heat (photothermal properties) have been reported, such as carbon nanomaterials, gold (Au) nanomaterials, metal oxides/sulfides, indocyanine green (ICG), dyes, melanin, and polyaniline.

**Figure 4:**
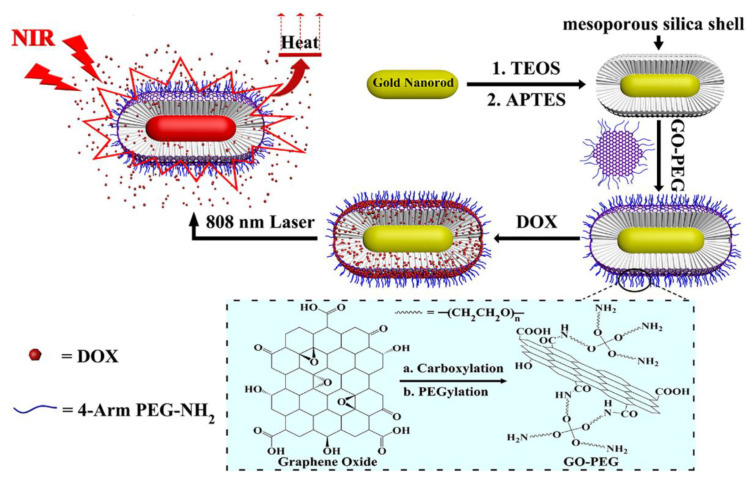
Preparation of GNRs/SiO_2_/GO-PEG nanoparticles. Reproduced from [36] with permission, copyright 2019, Elsevier.

Among these photothermal nano-agents, carbon nanomaterials, a type of converter with excellent photothermal conversion ability, such as graphene oxide (GO) and carbon nanotubes (CNTs), have shown several favorable properties, including strong NIR absorption, large surface area for drug encapsulation, easy surface functionalization, and low toxicity risk [68,69]. For instance, Qi et al. developed PEGylated GO-capped Au nanorods/silica nanoparticles loading DOX by stacking and electrostatic interactions (Figure 4). This nanovehicle exhibited high drug loading efficiency due to the large surface area of GO and outstanding photothermal conversion efficiency because of the synergistic photothermal effect of GO and Au nanorods. GO has functional groups such as free carboxylic and hydroxyl groups so that GO can bind to drugs through covalent bond, adsorption of drugs, hydrophobic attraction, and hydrogen bonding [36,70,71]. In addition, the functional groups of GO can enable effective targeted drug delivery by binding to the targeting moieties [72,73]. CNTs have several advantages such as chemical stability, robustness, several binding sites for targeting proteins, and penetrative ability through the cell membrane; they have additionally demonstrated their strong ability to transduce visible and NIR light to heat. Dei et al. recently created a novel DOX-loaded porous carbon nanofiber (DOX@PCNFs) that can release a drug payload under acidic conditions with NIR exposure [37]. The in vitro and in vivo results showed that the DOX@PCNFs exhibit high cellular uptake of the drug upon NIR light irradiation.

Metal nanoparticles such as Au, silver, platinum, and metal oxide/sulfide have been used as PTT materials because of their excellent ability to absorb light and produce heat [38,74,75,76,77,78,79]. Among the PTT materials, Au nanoparticles have been intensively studied for the suppression of tumors because of their ability to convert NIR light to heat by a strong localized surface plasmon resonance phenomenon in the NIR region [80]. For instance, Yang et al. reported a nanovehicle sensitive to NIR light and glutathione (GSH) that produced a chemo-photothermal synergistic effect. Au particles were conjugated with mesoporous silica (MPS) by a disulfide bond and DOX was encapsulated inside the pores of the MPS. These nanomaterials presented improved DOX and Au release resulting from the opening of pores through the cleavage of disulfide bonds in the presence of GSH and exhibited a synergistic effect in chemo-photothermal therapy under NIR irradiation [38]. Peng et al. developed a cupric sulfide (CuS)-based nanoplatform composed of CuS coated with MPS and DOX loaded in nanoparticles (CuS@MPS). The NIR thermal image of CuS@MPS-DOX showed great photothermal efficacy and synergistic effects in chemo-photothermal cancer therapy [39].

Recently, various organic dyes have attracted attention, and they have been encapsulated photo-responsive nanovehicles [81]. Although these organic dyes are limited by low stability under unceasing NIR light irradiation, they are potential converters for the future owing to their biodegradability. Among the organic dyes, Prussian blue (PB) and ICG have been studied. PB exhibits high NIR light absorption and biocompatibility [82], and ICG presents a strong ability to convert absorbed NIR light to heat and lesser toxicity and markedly decreases the effect time in the blood, considerably improving its efficacy in vivo owing to the quick elimination of ICG [83]. Cano-Mejia et al. showed photothermal immunotherapy, which combines PB nanoparticle (PBNP)-based PTT with anti-CTLA-4 checkpoint inhibition for treating neuroblastoma. PBNP reduced the tumor burden and enhanced the immune response, specifically, it increased intrusion of lymphocytes and T cells to the tumor site, which was complemented by the anticancer effects of anti-CTLA-4 immunotherapy, providing a more lasting treatment against neuroblastoma in in vivo experiments. Mice treated with photothermal immunotherapy showed protection against tumor rechallenge, resulting in improved immunity against tumors [40]. Yan and Qiu developed an ICG-based photothermal nanovehicle. They prepared ICG-encapsulated micelles with folate-conjugated poly(2-ethyl-2-oxazoline)-b-poly(ε-caprolactone) (FA-PEOz-PCL) [41]. The unstable ICG in water appeared to exhibit increased stability in micelles and hyperthermia effect under NIR irradiation. The in vivo data demonstrated that the micelles effectively targeted human epidermoid carcinoma cells (KB) tumor-bearing mice, indicating their potential for theragnostic applications in cancer.

#### 2.2.2. Photo-Responsive Nanovehicles Using PDT

PDT, a non-invasive cancer treatment that utilizes the generation of ROS via the reaction of photosensitizers (PSs) responding to appropriate light irradiation has attracted increased interest in the development of controlled targeting nanovehicles owing to their advantages of ease of control, non-invasiveness, and spatial control [84,85]. PDT using various PSs for cancer treatment has been well documented in other reviews [84,85,86]. Here, we briefly discuss photo-responsive nanovehicles by mediators induced by PSs via interaction with light illumination. Recently, nanomaterials encapsulating both PSs and drugs showed the feasibility of controlled drug release and increased effect on cancer treatment [87]. PSs absorb light energy to express ROS and deplete oxygen to induce hypoxia in cancer, enabling their application in mediator-responsive nanovehicles triggered by ROS or hypoxic conditions [88,89,90]. ROS-responsive nanovehicles using ROS originating from PSs under light radiation showed improved, controlled drug delivery through the decomposition of nanovehicles [42,91,92,93,94].

**Figure 5:**
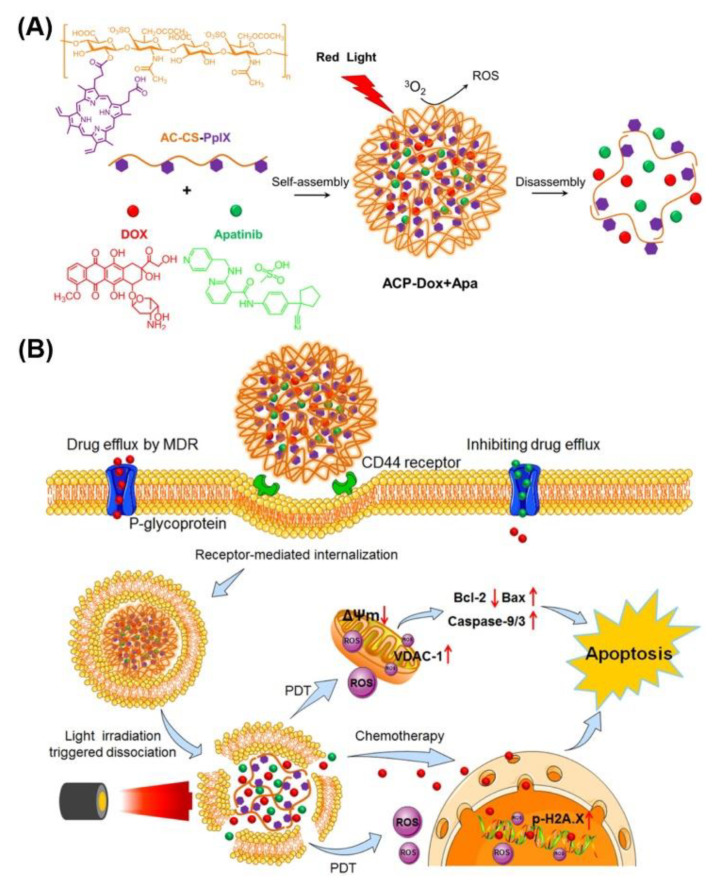
(**A**) Formation and decomposition of ACP-DOX + Apa micelles. (**B**) Underlying mechanism of combating the MDR (multidrug-resistant) using intelligent ACP-DOX + Apa micelles for promoting synergistic antitumor potency. Reproduced from [42] with permission, copyright 2018, American Chemical Society.

Drug release can be triggered in ROS-responsive nanovehicles only in ROS-rich cells or tissues when induced by highly reactive singlet oxygen generated from photo-responsive PSs. For example, Wei et al. designed a new ROS-responsive nanomedicine based on protoporphyrin IX (PpIX)-conjugated polymer micelles. The nanomedicine with acetylated-chondroitin sulfate (AC-CS) backbone was loaded with dual chemotherapeutics, DOX and apatinib (Apa), for the reversal of multidrug-resistant (MDR) tumors. When irradiated at 635 nm, the nanoplatform generated excessive ROS, triggering the release of DOX and Apa from the micelles by oxidation decomposition of the CS polysaccharide backbone. Subsequently, the released Apa competitively inhibited the P-glycoprotein drug transporter in the MDR tumor cell membrane, thereby leading to recovery of the chemical sensitivity of DOX, ROS overproduction, the PDT effect, and apoptosis (Figure 5) [42].

**Figure 6:**
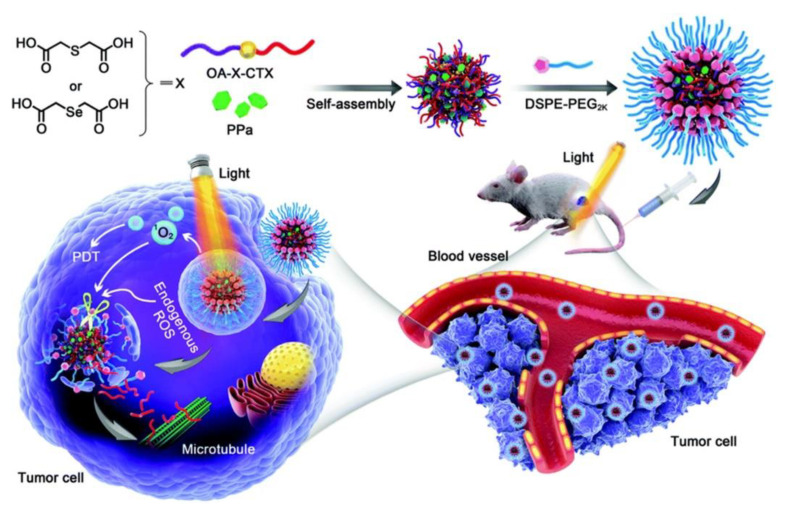
Schematic illustration of self-assembly of OA-X-PTX nanoparticle loaded with PPa and its cascade amplification release process. Reproduced from [44] with permission, copyright 2018, Royal Society of Chemistry.

ROS-triggered linker cleavage can also be exploited for controlled drug delivery with light exposure in ROS-activatable prodrug nanoplatforms [95,96,97]. ROS-activatable prodrugs are commonly composed of an ROS-trigger PS, drug, and ROS-responsive cleavage linker. The ROS-trigger PS generates ROS under light irradiation, the linker can be cleaved by endogenous ROS in the tumor or exogenous ROS from the ROS-trigger PS, and the drug can be released, causing a toxic effect. Bio et al. verified a novel approach to activate prodrugs with light using an ROS-cleavable prodrug in the mitochondria by PpIX [43]. Yang et al. prepared an ROS-activatable prodrug nanoplatform composed of 1,2-distearoyl-*sn*-glycero-3-phosphoethanolamine-*N*-[methoxy(polyethylene glycol)-2000] (DSPE-PEG2k) nanoparticles incorporated with pyropheophorbide a (PPa) and thioether/selenoether-linked conjugates of cabazitaxel (CTX) and oleic acid (OA) (Figure 6) [44]. In this nanoplatform, CTX was released not only through stimulation by ROS overexpressed in tumor cells, but also through ROS production by PPa when irradiated with external light. The ROS-responsive nanovehicles showed prolonged systemic circulation and drug accumulation in the tumor, demonstrating a synergistic antitumor effect.

**Figure 7:**
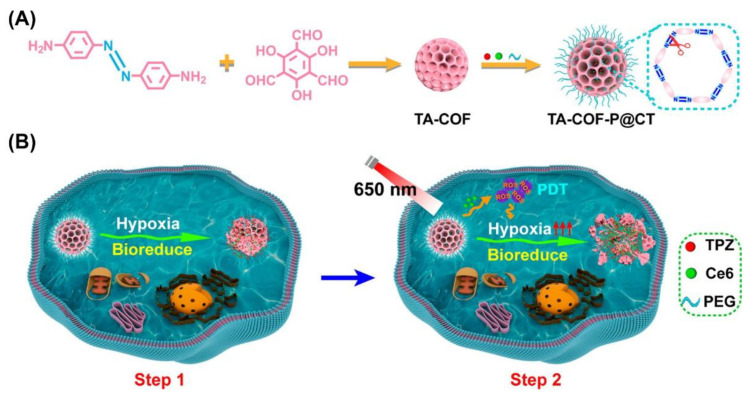
(**A**) Schematic illustration for the synthesis of TA-COF and TA-COF-P@CT. (**B**) Schematic illustration of two-step photo-activated hypoxia-responsive drug delivery toward cancer treatment. Reproduced from [46] with permission, copyright 2021, American Chemical Society.

In addition, hypoxic conditions have been used in nanovehicles based on photo-induced mediators. PDT was performed through light irradiation and continuously decreased oxygen generation, which resulted in a temporary hypoxic environment at the target sites. Hypoxia-responsive systems can detect and target tumor sites in a hypoxic environment [38,45,98,99]. Wang et al. designed a new delivery system that combines PDT with hypoxia-responsive nanovehicles. They synthesized the chlorine e6-PEG-azobenzene linked poly(caprolactone) (Ce6-PEG-Azo-PCL) by coupling Ce6-decorated PEG and PCL through hypoxia-responsive cleaved Azo linkage and loaded DOX into the nanovehicles prepared with the amphiphilic polymers. After the robust self-assembled nanovehicles were delivered to the tumor sites, light irradiation at 671 nm generated the ROS and hypoxia micro-environment through the activation of Ce6, which amplified the stepwise ROS- and hypoxia-triggered dissociation of Azo linkers through reduction and the release of DOX from the disassembled nanovehicles into the tumor cells. The PDT and hypoxia-responsive nanovehicles consequently showed integrated tumor suppression in vitro and in vivo [45]. In addition, to enhance anticancer efficiency, Ge et al. designed a photo-activated hypoxia-responsive prodrug loading covalent organic frameworks (COF) for the delivery of Ce6 and tirapazamine (TPZ) (TA-COF-P@CT) by combining PDT and chemotherapy. TPZ can be converted to cytotoxic radicals upon activation by various intracellular reductases under hypoxic conditions, such as in tumors. While the generated radicals were easily oxidized in a normal state with very few side effects, they were stable in a hypoxic environment formed by PDT, exhibiting anticancer effects in tumor cells. TA-COF-P@CT was prepared by the reaction of 1,3,5-triformyl-2,4,6-trihydroxybenzene (TP) and 4,4-azodiaminobenzene (AD), decorated with PEG, and co-loaded with TPZ and Ce6. The ROS generated by light (650 nm) irradiation created a hypoxic environment by consuming oxygen, decomposed COF by breaking the azo linkage via overexpressed azo reductase in tumor cells under hypoxic conditions, and released the loaded Ce6 and TPZ to kill cancer cells by generating biotoxic oxyradicals (Figure 7) [46].

Although controlled drug release using PDT has been demonstrated, photodynamic responsive nanovehicles present certain challenges that need to be overcome. Endogenous ROS or hypoxia in biological systems can lead to inappropriate drug release and side effects. In addition, the PSs in these PDT systems are usually organic dyes that have poor optical stability upon light irradiation [100]. Hence, the development of PSs with enhanced photostability is an important step for enabling their use in broad applications.

## 3. pH-Responsive Nanovehicles

pH-responsive nanovehicles have been intensively exploited among environmental stimuli-responsive nanovehicles, since it was discovered that the extracellular pH near tumors is more acidic than that in normal tissues [24]. Hydrogen ions (called protons) in acidic conditions can affect the structure of nanosized nanovehicles, resulting in pH responsiveness. Here, two types of pH-responsive nanovehicles are discussed; de/protonation-based nanovehicles and acid-labile bond cleavage-based nanovehicles.

### 3.1. De/Protonation-Based Nanovehicles

De/protonation is the most commonly used mechanism for pH-responsive nanovehicles in cancer therapy. As shown in Table 2, pH-responsive nanovehicles typically include polyelectrolytes, such as cationic poly(β-amino ester) (PBAE), PDMAEMA, poly(histidine) (poly(His)), poly(aspartic acid-graft-imidazole) (poly(Asp-g-im)), and anionic poly(Asp), poly(acrylic acid) (PAA), polysulfonamide, etc. For advanced strategies to develop biodegradable polyelectrolytes, it was also reported that biodegradable polymers such as polypeptides and enzyme-sensitive crosslinked chitosan were utilized for de/protonation-based nanovehicles by conjugating with pH-sensitive moiety to the biodegradable polymer backbones [101,102,103]. The polyelectrolyte usually includes amine groups as cationic moieties and –COOH as anionic moieties blocked with other polymers, such as hydrophilic or hydrophobic polymers, which have been further utilized in pH-responsive polymers using protonation and deprotonation mechanisms.

The cationic polyelectrolytes with amine groups, including PEG-poly(β-amino esters)-poly lactic acid (PLA), PEG-poly(2-(diisopropylamino) ethyl methacrylate), 1,2-distearoyl-*sn*-glycero-3-phosphoethanolamine-*N*-[methoxy(polyethylene glycol)] conjugated poly(β-amino esters), PEG-poly(2-(diisopropylamino) ethyl methacrylate-co-dithiomaleimide), PEG-poly(2-(dibutylamino) ethyl methacrylate-co-dithiomaleimide), and poly(N-vinylpyrrolidone)-poly(4-vinylpyridine) [104,105,106,107,108], can protonate under acidic conditions showing hydrophilicity, while they can deprotonate under basic conditions, indicating hydrophobicity (_−_NR_2_ ↔ NR_3_^+^). In contrast, anionic polyelectrolytes with –COOH, such as poly(N-isopropylacrylamide-co-acrylic acid), PCL-SS-poly(methacrylic acid), CTS-poly(methacrylic acid-co-N-isopropylacrylamide), poly(*N*-(4-methacrylamido)-*N*-(4,6-dimethylpyrimidin-2-yl)benzene-1-sulfonamide-co-*N*,*N*′-dimethylacrylamide) [109,110,111,112], can deprotonate and protonate in the opposite manner. For example, imidazole groups with a pair of electrons on the unsaturated nitrogen atom can be easily protonated in slightly acidic environments, resulting in conversion from hydrophobic to hydrophilic [113,114,115,116,117,118,119,120,121]. This can cause destabilization of the nanovehicles and consequently release the encapsulated drug. Poly(His)-PEG, developed by Bae et al., showed robust nano-sized core-shell micelles at physiologic pH composed of hydrophobic cores by deprotonation of poly(His) and the hydrophilic shell of PEG. However, at pH_ex_, the protonation of poly(His) was triggered in His moieties and induced the rupture of micelles due to the decrease in poly(His) hydrophobicity. Furthermore, the hydrophobic anticancer drugs incorporated in the core of the pH-responsive micelles could be released under acidic conditions (pH_en_ or pH_ex_) owing to the formation of a less hydrophobic core [117]. In addition, Oh’s group synthesized poly[(benzyl-*L*-aspartate)-co-(N-(3-aminopropyl)imidazole-*L*-aspartamide)]-PEG (PABI-PEG) for docetaxel (DTX) delivery [122]. PABI-PEG formed a stable nanovehicle at pH 7.4 or higher; however, in acidic conditions, it became unstable due to protonation of the imidazole group. DTX-loaded micelles showed pH-responsive drug release due to structural changes caused by protonation of the imidazole group on the PABI blocks. pH-responsive drug release and very low micelle concentrations at physiological pH can result in high stability and reduce the toxicity of normal tissues, limiting drug loss.

Anionic polyelectrolytes have also been utilized for pH-responsive nanovehicles to target tumors. The strategy using anionic amphiphilic block copolymer for tumor targeting and pH-responsive nanovehicles can be different from that using cationic polymers. At a low pH, such as pH_en_ and pH_ex_, anionic polymer blocks including –COOH can exist as protonated (hydrophobic) blocks and cannot be used in tumor-targeting micelles from anionic amphiphilic block copolymers. Therefore, the anionic block copolymer can be coupled with basic drugs such as DOX using electrostatic interactions at physiological pH, and the drug can be released at acidic pH through reduced interaction due to protonation. For example, Yi et al. reported anionic block copolymers composed of PEG, PCL, and carboxyl-modified PCL (COOH-PCEC) [123]. These copolymers encapsulated DOX through electrostatic and hydrophobic interactions. The release of DOX was faster under acidic conditions than under neutral conditions.

### 3.2. Acid-Labile Bond Cleavage-Based Nanovehicles

As mentioned previously, the differences in pH among the intracellular compartments and between normal tissues and tumors have attracted interest for the development of pH-dependent chemical structures. In particular, acid-labile bonds have been intensively studied for triggering pH-responsive nanovehicles in pH_ex_ or pH_en_. Labile structures such as hydrazone, imine, acetal, ester, and amide can be cleaved by acid hydrolysis in protic acid as a catalyzer via a nucleophilic substitution reaction. The acid-labile linkers are stable in normal tissues (pH ~7.4) but are breached in the acidic micro-environment of the tumor by hydrolysis. Acid-labile chemicals have been used as functional groups in nanoplatforms and are usually linked directly to the anticancer agents. Table 3 shows the most investigated pH-responsive chemical bonds in cancer treatment and their degradation products.

#### 3.2.1. C=N Bonds (Carbon–Nitrogen Double-Bonds)

Labile chemicals containing C=N, such as hydrazones, imines, and oximes, with their protonation in the sp2 nitrogen of the bond in an acidic environment, can be highly susceptible to nucleophilic attack by water due to the enhanced electrophilicity of the sp2 carbon [137]. In particular, hydrazone linkage with higher sensitivity at pH_en_ (pH 5.0) and a faster hydrolysis rate has been popularly applied to various pH-responsive systems such as micelles, liposomes, dendrimers, linear polymers, star-shaped polymers, and inorganic nanoparticles [124,138,139,140,141,142]. In addition, acid-labile bonds have been used to address the PEGylation problem, in which the hydrophilic PEG coating of nanovehicles limits drug release from the nanovehicle core and interferes with target-cell interactions and endosomal escape. For instance, Manju Kanamala et al. used a hydrazone linker to solve the PEG dilemma through cleavable PEGylation. They synthesized a PEG-cleavable pH-responsive liposome (CL-PEG-pSL) and studied the feasibility of the PEG-detachment strategy in the micro-environment of cancer cells. Compared to general liposomes, CL-PEG-pSL showed improved endo/lysosomal escape ability in cancer cells and high tumor accumulation in the MIA PaCa-2 pancreatic cancer cell xenograft model [125].

**Figure 8:**
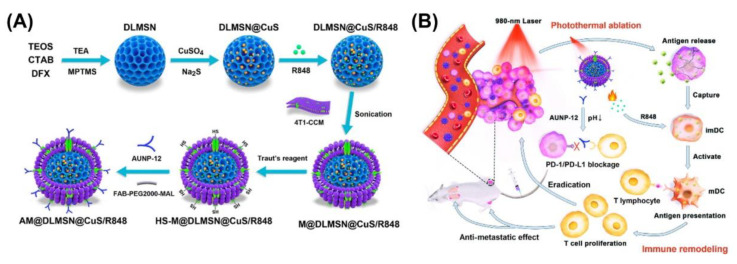
(**A**) Schematic illustrations for the preparation and (**B**) synergistic effects of AM@DLMSN@CuS/R848 against triple-negative breast cancer (TNBC) by combining photothermal ablation and immune remodeling. Reproduced from [144] with permission, copyright 2020, American Chemical Society.

Imine bonds, unlike hydrazone, showed low stability at physiological pH due to the absence of a mesomeric effect [137]. Accordingly, research has been conducted to increase the stability by introducing π–π junctions with structures such as benzoic imine and poly (propylene imine) [126,127,143]. Yuanyuan et al. designed a nanoplatform based on dendritic large-pore mesoporous silica nanoparticles (DLMSNs) conjugated to peptides via benzoic imine bonds using formyl benzoic acid-PEG-maleimide. After encapsulation of CuS nanoparticles and immune adjuvant resiquimod (R848) in DLMSNs, the anti-PD-1 peptide AUNP-12 was conjugated to the surface through an acid-labile benzoic imine bond. The pH-responsive nanoplatform released AUNP-12 through cleavage of the imine bond at a weakly acidic pH_ex_ 6.5 and showed excellent PD-1/PD-L1 blocking efficacy. When subjected to 960 nm laser irradiation, the systems induced photothermal ablation, resulting in synergistic tumor vaccination and T lymphocyte activation, preventing tumor recurrence and metastasis (Figure 8) [144].

Additionally, oxime linkers with several advantages, such as click chemistry, high stability, chemical selectivity, and compatibility with the functional groups of biomolecules [128,145,146], have been researched for the development of pharmaceutical applications using acid-labile bonds. Eirinaios et al. developed a prodrug GOXG, which is a rapid and cost-effective “click” oxime bond ligation platform to assemble in one-pot peptide-drug conjugates (PDCs). PDCs with the anticancer drug gemcitabine and D-Lys6-GnRH (gonadotropin-releasing hormone; GnRH) as a cancer-targeting material induced the separation of drugs from GOXG at pH_en_ and pH_lys_ through breakage of the acid-labile oxime bond. [129].

#### 3.2.2. Other Acid-Labile Bonds

Acetals and ketals are stable under basic conditions; however, they are easily hydrolyzed to aldehydes, ketones, and alcohols in an acidic environment. Both undergo first-order hydrolysis of hydronium ions, and the rate of hydrolysis can increase by 10 times as the pH decreases [130]. Polyketals, which are more sensitive to pH than hydrazone, are hydrophobic polymers with biodegradable ketal bonds in the polymer backbone; they can encapsulate hydrophobic drugs or proteins [131,147,148]. In addition, these chemical bonds can be used with other reaction systems to produce better results [132].

Since amides, as derivatives of carboxylic acids, are highly stable, strong acidic or strong alkaline conditions are required to hydrolyze them. Considering that amides can be degraded in the acidic tumor micro-environment, research on maleic acid amides has been in the spotlight. Maleic acid derivatives can exhibit high pH sensitivity because adjacent carboxylate groups easily attack the carbonyl group of the amide to form a tetrahedral intermediate with a 5-membered ring [149]. Furthermore, researchers have used substituted amide linkages such as β-carboxylic amides and *cis*-aconityl amide for tumor targeting [133,150]. The *cis*-aconityl amide linker undergoes acid-catalyzed hydrolysis at a hydrolytic bond (C-1) bond, leading to more complete drug release at the target because of the high acid lability compared to the *trans* form [151]. β-carboxylic amides maintain a negative charge at physiological pH 7.4; however, they transform into positively charged primary amines under the acidic pH in the tumor, which results in a rapid drug release and improved cell transduction efficiency due to electrostatic absorption endocytosis [133].

**Figure 9:**
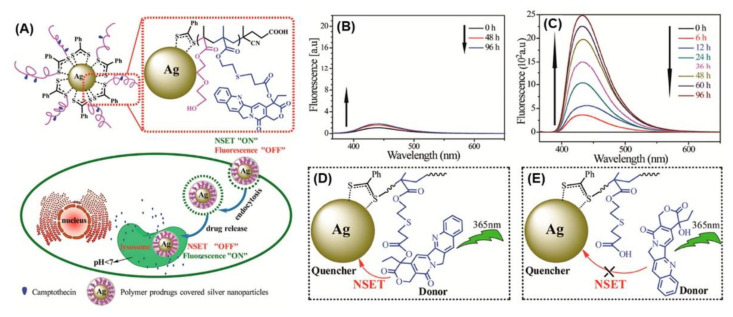
(**A**) Schematic illustration and intracellular release of CPT. Principle of NSET (nanoparticle surface energy transfer) “on” and “off” for the P(HEO2MA-co-MACPT)@AgNPs. Fluorescence spectra of the hybrid AgNPs after incubation in PBS solution at pH 7.4 (**B**) and at pH 5.0 (**C**) for different times. Schematic diagrams of NSET “on” (**D**) and NSET “off” (**E**) for the P(HEO2MA-co-MACPT)@AgNPs. Reproduced from [154] with permission, copyright 2017, American Chemical Society.

β-thiopropionate, which contains ester bonds, including succinic ester bonds, undergoes hydrolysis under both acidic and alkaline conditions. The succinic ester can be formed by the reaction of the linking unit succinic acid, which is composed of two carboxyl groups and a hydroxyl group [152,153]. Compared to other acid-responsive bonds, the β-thiopropionate formed by the linking thiol and acrylate can be hydrolyzed in an acidic solution at a relatively slow rate to apply for sustained release of drugs [135,136]. Qiu et al. developed a switchable fluorescent “Off” or “On” silver nanoparticle (AgNP) through the nanoparticle surface energy transfer (NSET) effect. The hybrid nanoplatform (P(HEO_2_MA-co-MACPT)@AgNP) was prepared by conjugating with poly(methacryloyloxy-3-thiahexanoyl camptothecin (CPT)-co-2-(2-hydroxyethoxy)ethyl methacrylate) P(HEO_2_MA-co-MACPT) and AgNPs using β-thiopropionate bonds. The NSET effect is a spectral phenomenon in which electronically excited “donor” molecules (such as fluorescent molecules) transfer excitation energy to nanoparticles depending on the distance between the donor and acceptor. They used the acid-labile β-thiopropionate to control the NSET phenomenon by varying the physical distance between camptothecin (CPT) and AgNPs. At pH 7.4, CPT fluorescence was dissipated due to the NSET effect because the polymer backbone kept the distance between CPT and AgNP close. In an acidic environment, the fluorescence was recovered because the β-thiopropionate bond was cleaved; subsequently, CPT was released from the nanovehicle. Additionally, the intensity of fluorescence increased over time owing to the gradual decomposition of acid-labile bonds. The cytotoxicity of the CPT-loaded nanovehicles showed pH-dependent effects and exhibited the potential for use in studying the mechanisms of drug release behavior in cells based on changes in fluorescence (Figure 9) [154].

## 4. Photo- and pH-Dual-Responsive Nanovehicles

Most stimuli-responsive nanovehicles have been designed using one stimulus; however, the biological performance of macromolecules is responsive to multiple stimuli, resulting in several changes. For mimicking biological processes, various stimuli-responsive moieties can be incorporated into a single nanovehicle, creating multi-stimuli-responsive materials to provide more than one mechanism responsiveness for targeting cells [155,156,157]. The purpose of multi-stimuli-responsive nanovehicles is to achieve long circulation, high accumulation in targeted sites, deep penetration in targeted tissues such as tumors, internalization in targeted cells, endosome escape, and controlled drug release. [157,158,159,160,161,162,163,164]. In addition, multi-stimuli-responsive nanovehicles have been engineered to facilitate multistage drug delivery and achieve higher specificity and efficacy [157]. Recently, dual-responsive nanovehicles that use light and pH responsiveness have been widely studied. Nanovehicles can be fabricated using materials including polymers, liposomes, and solid inorganic nanoparticles [165]. Various reactions to multi-stimuli-responsive nanovehicles have been observed, such as charge conversion (e.g., de/protonation), change of structure/shape or size conformation (e.g., degradation/cleavage/breakage) of the nanovehicles, and sol-gel transition [155,157,166]. Among them, we focus on photo- and pH-dual-responsive nanovehicles using the mechanisms of de/protonation and cleavage of the nanovehicles, as previously mentioned.

### 4.1. De/Protonation Triggered by Light- and pH-Dual-Responsive Nanovehicles

Dual stimuli-responsive nanovehicles using pH-responsive polymers and incorporating PSs showed charge conversion such as protonation and deprotonation of the nanovehicles in acidic or basic conditions and increased effects of PTT or PDT under light irradiation. For example, Oh’s group developed pH-responsive polymers based on imidazole-modified polypeptides for cancer targeting. They fabricated an on-demand pH-sensitive nanocluster (NC) system encapsulating PS, gold nanorods (AuNRs), and DOX in a pH-responsive polymer, poly(aspartic acid-graft-imidazole)-PEG (PAIM-PEG), to improve the therapeutic effect of chemo-photothermal therapy [167]. The NC system sustained a firm nano-assembly, structured with less systemic toxicity at pH 7.4; they formed disintegrated structures due to destabilization of their hydrophobic cores by protonation of the imidazole rings and carboxyl groups in PAIM-PEG and released higher amounts of the drug at pH 6.5. Additionally, the NC enhanced antitumor efficacy synergistically, resulting from the improved accumulation and release of DOX from the NC system and PTT of Au under locally irradiated NIR light. In another study, Oh’s group developed visible light- and pH-responsive nanovehicles using PAIM-PEG and a photosensitive agent, indole-3-acetic acid (IAA), for cancer treatment. Researchers have reported that protonation of the imidazole and carboxyl groups of PAIM-PEG, resulting in destabilization of the micelle structures at acidic pH, induced a synergistic ROS generation from IAA upon irradiation with visible light. At physiological pH, lower systemic toxicity was observed in IAA-loaded micelles (ILMs). Interestingly, the increasing accumulation and release of IAA from the micelles at pH_ex_ or pH_en_ and upon simultaneous local irradiation of visible light resulted in maximizing antitumor efficacy, even when the amount of IAA was less than the IC_50_ of IAA [168]. More interestingly, the utilization of visible light instead of UV light could be expected to decrease the side effects of UV light in clinical applications.

**Figure 10:**
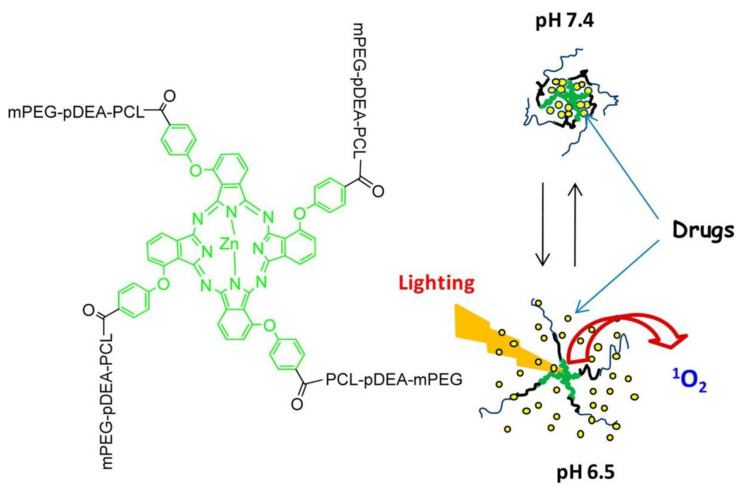
Molecular structure of PDCZP and illustration of its pH-dependent drug release and combination of chemotherapy and photodynamic therapy. Reproduced from [169] with permission, copyright 2017, American Chemical Society.

In addition, various structures such as star-shaped polymers and dendrimers have been highly utilized to construct dual stimuli-responsive nanovehicles. Zhang et al. constructed a PS core 4-armed star-shaped copolymer composed of [PEG-poly(2-(*N*,*N*-diethylamino)ethyl methacrylate) (pDEA)-PCL]_4_-zinc β-tetra-(4-carboxyl benzyloxyl)phthalocyanine (PDCZP) capable of targeting tumors and responding to dual stimuli, light, and pH. The pH responsiveness of PDCZP resulted from pDEA chains, which could shrink in weakly basic environments (pH 7.4) through hydrophobic interaction and, in contrast, extend in weakly acidic environments (pH 6.5 or 5.0) because of increased hydrophilicity due to protonation of the amines of pDEA. The DOX-loaded nanovehicles showed the formation of 50 nm-sized spherical particles at pH 7.4. In the nanovehicles delivered to tumor cells, the rapid DOX release was triggered by the acidic pH, resulting in enhanced antitumor effects through chemotherapy with DOX and PDT with core PS under light irradiation (Figure 10) [169].

**Figure 11:**
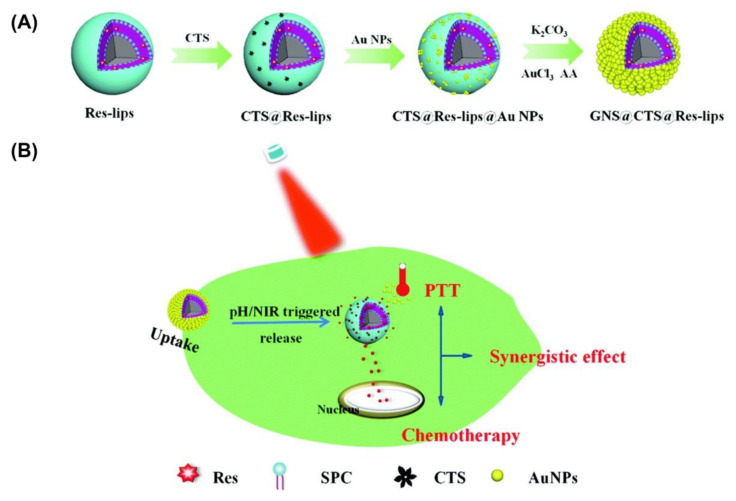
(**A**) Schematic representation of the synthetic strategy for GNS@CTS@Res-lips. (**B**) GNS@CTS@Res-lips as pH/NIR dual-responsive drug deliveries for synergistic chemo-photothermal cancer therapy. Reproduced from [170] with permission, copyright 2017, Royal Society of Chemistry.

Yuan et al. designed dual-responsive dendrimers containing SP groups for photo- and pH-responsive nanovehicles. The star-shaped dendrimers were prepared by the conjugation of dendritic polyester and poly(ε-caprolactone)-poly(methacrylic acid-co-spiropyran methacrylate) (DPCL-b-P(MAA-co-SPMA)). In this system, the isomerization of SP groups under UV light irradiation or low pH resulted in either light- or pH-responsive abilities. The SP isomerized to hydrophilic merocyanine MC under light irradiation and changed to merocyanine H+ (MCH) through protonation upon acid addition. These results showed that the DOX-loaded dendrimers exhibited drug release when triggered by UV irradiation or under acidic conditions and that, consequently, the controlled release system based on SP was developed by either adjusting UV/Vis light illumination or changing the pH values [158]. In addition, Wang and co-workers designed and prepared chitosan (CTS)-modified liposomes loaded with resveratrol (Res) and coated them with Au nanoshells (GNS@CTS@Res-lips). The drug release from the GNS@CTS@Res-lips, caused by pH- and photo-dual-responsiveness, prominently increased the drug cellular uptake and chemo-photothermal effect under NIR light irradiation (Figure 11). This study showed that the higher release of Res at pH 5.0 (vs. pH 7.4) might be caused by protonation of amino groups in CTS molecules in an acidic environment, which weakens the electrostatic interaction with CTS and phospholipids, resulting in easy diffusion of Res molecules from the liposomes [170].

### 4.2. Degradation/Cleavage/Breakage Triggered by Light- and pH-Dual-Responsive Nanovehicles

**Figure 12:**
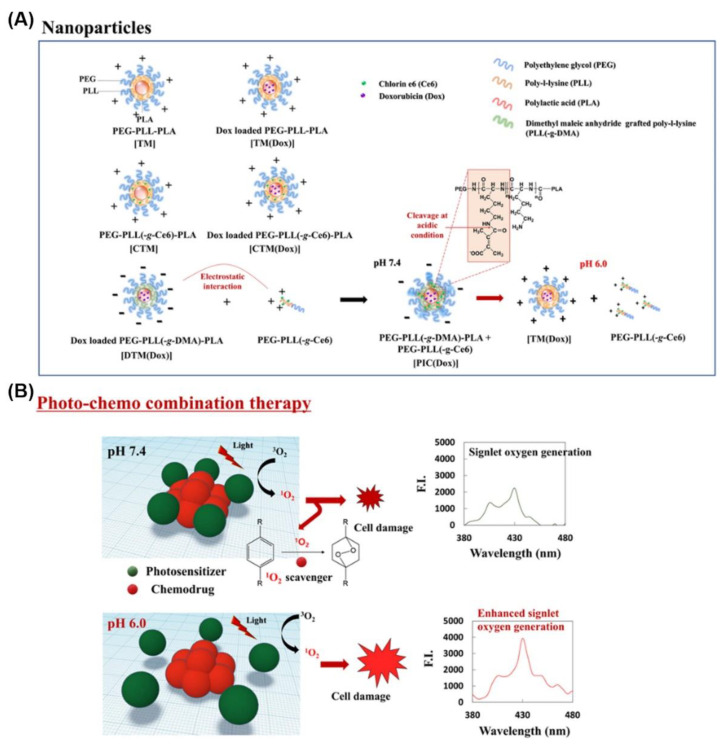
(**A**) Prepared drug loading nanoparticles. (**B**) Photosensitizer and chemo-drug interaction in the process of generating singlet oxygens. Reproduced from [171] with permission, copyright 2019, Elsevier.

Many studies have reported the utilization of light- and pH-responsive systems for triggering the degradation/cleavage/breakage of nanovehicles. Oh’s group researched polyelectrolyte nanoparticles composed of [PEG-2,3-dimethylmaleic anhydride grafted poly(l-lysine)-poly(lactic acid)] PEG-PLL(-g-DMA)-PLA [168]. They developed a charge-reversible nanovehicle using PEG-PLL(-g-Ce6, DMA)-PLA for PDT (Figure 12). The DMA linkage to the lysine residue could be cleaved in response to a decrease in the pH of the buffer, thereby regenerating the positive charge. The nanovehicle formed a stable structure owing to the hydrophobic interaction of PLA and showed surface charge conversion at acidic pH, which improved cell absorption, resulting in increased photo-toxicity. In addition, they studied various anticancer therapies using the surface charge conversion properties of PEG–PLL(-g-DMA)–PLA. For example, they developed a novel pH-responsive poly ionomer complex system composed of PEG-PLL(-g-Ce6) and PEG-PLL(-g-DMA)-PLA. The poly ionomer complex (PIC) system modulated the distance between the PS and DOX to resolve the antagonistic effect of reducing the singlet oxygen as the distance between the two materials reduced. This system exhibited improved single anti-oxygen production and anti-tumor activity compared to conventional nanovehicles because of changes in the distance in the PIC system under acidic conditions [171].

**Figure 13:**
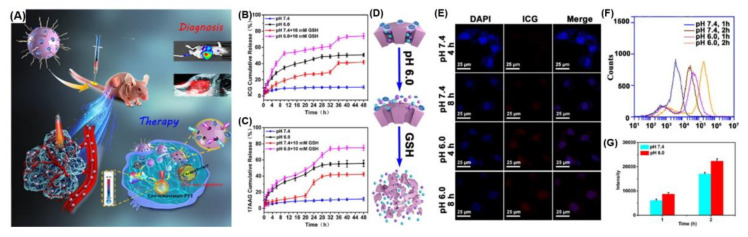
(**A**) Graphical abstract of chemo-drug-gated biodegradable hollow mesoporous organosilica nanotheranostics. In vitro ICG (**B**) and 17AAG (**C**) release profiles from ICG–17AAG@HMONs–Gem–PEG at different pH values with or without addition of 10 mM GSH. (**D**) pH-induced hydrolysis of acetal bonds and biodegradation of the HMON (hollow mesoporous organic silica nanocapsules) framework, triggering the pH/GSH-responsive payload release from the nanoplatform. (**E**) Confocal fluorescence images of MDA-MB-231 cells treated with ICG–17AAG@HMONs–Gem–PEG at pH 6.0 or 7.4 for 2 or 4 h. Scale bar: 25 μm. (**F**) Flow cytometry histogram profiles of intracellular fluorescence of ICG–17AAG@HMONs–Gem–PEG and (**G**) quantitative mean fluorescence intensities of the treated MDA-MB-231 cells at pH 7.4 and 6.0, respectively. Reproduced from [172] with permission, copyright 2018, American Chemical Society.

In a recent study, Wu et al. studied a cap removal strategy using acetal bonds for multimodal imaging-guided low-temperature PTT/chemotherapy of cancer. They encapsulated ICG and the Hsp90 inhibitor 17AAG in hollow mesoporous organic silica nanocapsules (HMONs) and subsequently blocked them with gemcitabine (Gem) molecules through acetal covalent bonds. At pH 7.4, small amounts of ICG and 17AAG were released, confirming the excellent capping effect of Gem under physiological conditions. In contrast, the release of ICG and 17AAG increased dramatically at pH 5.0. The investigation of cellular uptake and intracellular drug release using a laser confocal scanning microscope showed strong intracellular ICG fluorescence at pH 6.0 but relatively weak fluorescence at pH 7.4. These results indicated that ICG release was greatly stimulated by cleavage of the acetal bond. In quantitative flow cytometric measurements, the cellular uptake of the nanoplatform increased dramatically as the cell culture pH was changed from 7.4 to 6.0. This study presented a nanoplatform applying a pH-responsive gatekeeper, which minimizes damage to normal cells and provides an excellent low-temperature PTT strategy for cancer cell inhibition (Figure 13) [172].

**Figure 14:**
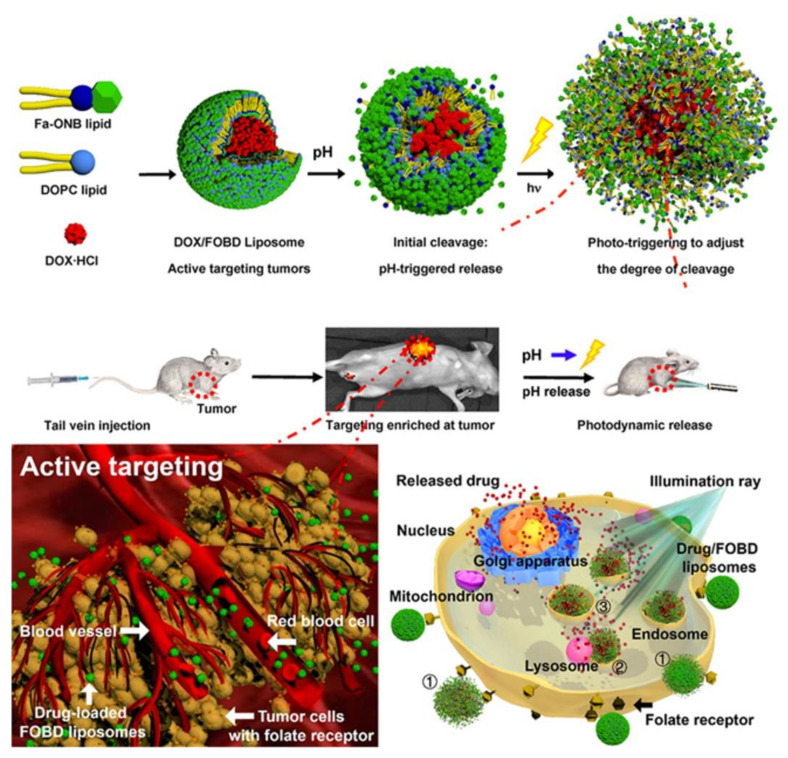
Schematic depiction of FOBD liposomes for active targeting and controlled drug release. Reproduced from [173] with permission, copyright 2020, American Chemical Society.

Qiao et al. designed a pH- and photo-dual-responsive multifunctional lipid (Fa-ONB lipid) consisting of activated folic acid combined with 4-(bromomethyl)-3-nitro-benzoic acid and a didodecylamine scaffold. The Fa-ONB lipid could be cleaved by both an acidic environment and UV light irradiation because of the *o*-nitrobenzyl ester bond. DOX-loaded liposomes composed of Fa-ONB lipid and dipalmitoylphosphatidylcholine (FOBD) showed an increase in drug release efficiency according to pH change and UV irradiation synergistically (Figure 14) [173]. Nisar et al. engineered a photocleavable and pH-responsive crosslinked nanovehicle. The hydrogel was prepared by functionalized CTS and a photocleavable crosslinker, 4-formylphenyl 4-((4-formylphenoxy)methyl)-3-nitrobenzoate (CHO–ONB–CHO). The hydrogel displayed not only a pH-responsive release behavior at acidic pH but also a photocleavable behavior of the crosslinker when absorbing the UV light (310–340 nm). These properties enabled drug release via hydrogel degradation [174].

**Figure 15:**
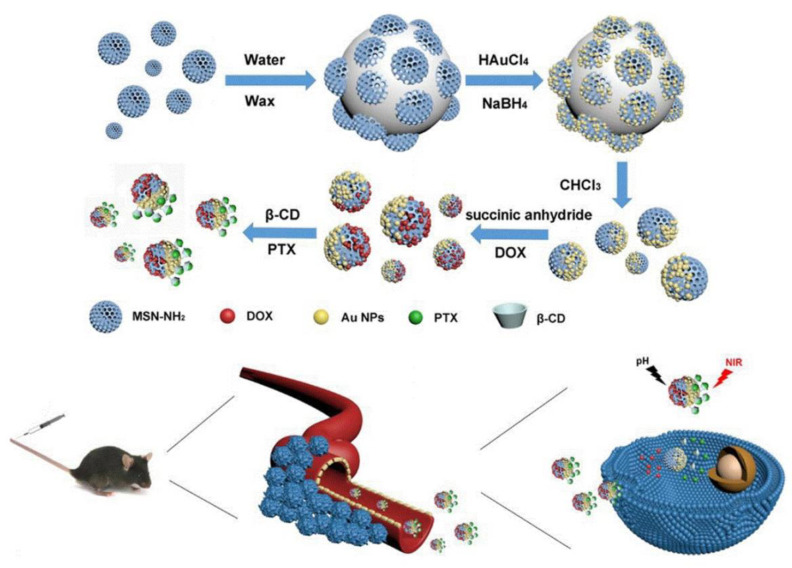
Schematic illustration of the preparation of PTX-Au-MSN-DOX JNPs by the Pickering emulsion method and their applications in vitro and in vivo. Reproduced from [176] with permission, copyright 2020, American Chemical Society.

Knežević et al. constructed a photo- and pH-dual-responsive nanovehicle using nitroveratryl-carbamate-protected aminopropyl-functionalized MSNs. DOX was adsorbed on nitroveratryl-carbamate-protected aminopropyl-functionalized MSNs. The photo-cleavage of the carbamate linkages yielded the release of the drug from the nanovehicle. Under UV light irradiation, positively charged propylammonium groups were generated on the nanoparticle surface, leading to the desorption of positively charged DOX from the surface of the nanoparticles. An increase in DOX release was under a weakly acidic environment and prolonged irradiation time [175]. Xing et al. synthesized Janus Au-MSNs loaded with paclitaxel (PTX) and DOX (PTX-Au-MSN-DOX JNPs). The PTX-Au-MSN-DOX JNPs exhibited pH and NIR dual-responsive release properties. This system used thiol-β-cyclodextrin as a vehicle for PTX on gold domains, while the other MS part served as a vehicle for DOX. The pH-sensitive DOX release occurred because of the protonation and dissociation of their amine groups under acidic conditions, whereas the release of PTX was caused by the breakage of the Au–S bond under NIR irradiation (Figure 15) [176]. Lu et al. developed nanovehicles using a new generation of hollow MSNs (HMSNs) to address the issue of low drug loading in traditional MSNs. HMSNs were prepared to encapsulate rose bengal (RB) and DOX. The surface of HMSNs was modified with hyaluronic acid (HA) via pH-sensitive Schiff base bonds (RB-DOX@HMSNs-N=C-HA). The pH-responsive Schiff base bonds were designed to be hydrolyzed under acidic conditions, leading to DOX and RB release from HMSNs and inhibition of tumor cell viability under light illumination [173]. In a recent study, they fabricated a targeted HMSN-based DDS for cancer chemo-PDT. ICG and DOX were co-loaded into HMSNs. Dopamine-modified hyaluronic acid (DA-HA) was connected to the HMSNs through boronate ester bonds for blocking the mesoporous channels of the HMSNs (ID@HMSNs-B-HA). Boronate ester bonds are acid-sensitive bonds that can exist stably under alkaline conditions and break in a weakly acidic environment. DA-HA can wrap HMSNs well under physiological conditions while detaching from the surface of HMSNs under acidic conditions, resulting in drug release [177].

## 5. Conclusions

In this review, we address the mechanisms and design of photo-responsive nanovehicles on the basis of recent research and the properties of polymers, peptides, and chemical groups responsive to the tumor acidic micro-environment. Several materials that absorb light can change their structures or release heat and ROS directly or indirectly through PSs, thus exhibiting a great potential for constructing photo-responsive nanovehicles for drug delivery. In addition, various materials with pH sensitivity and acid-labile chemical bonds can be used in nanovehicles depending on their properties. Such smart nanovehicles can improve the targeting of anticancer drugs and enhance tumor intracellular accumulation and uptake, resulting in increased anticancer efficacy and reduced systemic side effects. Recently, the development of two or more stimulus-based smart nanovehicles has been eagerly supported. In the future, it will be possible to obtain better results for cancer treatment owing to these multifunctional nanovehicles. Despite the wide range of efforts in this new direction, there remain several challenges in improving the therapeutic efficacy and safety of smart nanovehicles for clinical applications [178,179,180].

Photo-based transformation has critical drawbacks, such as low sensitivity to NIR light and relatively high optical power density for most reactive motifs. Indirect photo-responsive delivery may have disadvantages such as hypoxia-boosted tumor metastasis and ROS or heat-induced damage to the drug encapsulated in the carrier. The acidic pH region of tumors is generally far from the bloodstream, which may lead to an insufficient response of acidic-pH-responsive nanoparticles. Additionally, as shown in Scheme 2, the pH difference between healthy tissue and tumor tissue is not significant [181,182]. If future research is focused on this area and is directed toward a solution, the photo- and/or pH-induced nanovehicles can considerably benefit chemotherapy.

## Figures and Tables

**Table 1:** Summary of representative photo-responsive nanovehicles.

Classification	DDS	Photosensitive Material	Wave Length	Tumor Model	Ref.
Photo-Induced Chemical Transformation					
Photo- Isomerization	Photo-responsive cationic vesicle	Azobenzene	350 nm, 434 nm	MDA-MB-231	[32]
	Micelle based on SP-(PDMAEMA-block-PMMA), SP-(PMMA-block-PDMAEMA) blocks	Spiropyran	365 nm	HeLa	[33]
Photo-Induced Cleavage	MCP/DOC/shRNA	Coumarinyl ester	405 nm, 365 nm	HepG2/ADR	[34]
	LIP-DT-COU-MTX	Coumarin	800 nm	HeLa, A549	[35]
Photo-mediated materials					
Photothermal Therapy (PTT)	GNRs/SiO2/GO-PEG	Graphene oxide	808 nm	MCF-7	[36]
	DOX@PCNFs	Carbon nanotubes	808 nm	Mg-63	[37]
	DOX/MSN–Au	Au	808 nm	A549	[38]
	CuS@MPS-DOX	Cupric sulfide	808 nm	U87MG	[39]
	PBNP	Prussian blue	808 nm	Neuro2a	[40]
	FM	Indocyanine green	808 nm	KB	[41]
Photodynamic Therapy (PDT)	ACP-DOX + Apa	Protoporphyrin IX	635 nm	MCF-7/ADR	[42]
	Rh-L-CA4	Protoporphyrin IX	531 nm	AY-27	[43]
	PPa@prodrug NPs	Pyropheophorbide a	660 nm	4T1	[44]
	Ce6-PEG-Azo-PCL	Chlorine e6	671 nm	HeLa	[45]
	TA-COF-P@CT	Chlorine e6	650 nm	4T1	[46]

**Table 2:** Examples of pH-sensitive cationic and anionic polymers.

Type	Polymer	Chemical Structure	p*K*_a_
Cationic Polymers	Poly(β-amino ester)	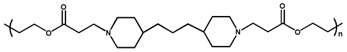	6.50
	Poly(2-(diisopropylamino) ethyl methacrylate)	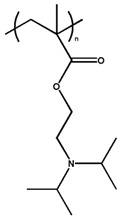	6.20
	Poly(histidine)	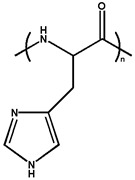	~7.0
	Poly(aspartic acid-graft-imidazole)	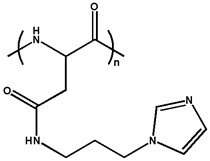	6.50
	Poly(4-vinylpyridine)	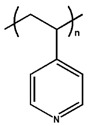	5.62
Anionic Polymers	Poly(aspartic acid)	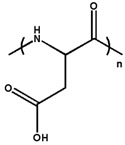	4.88
	Poly(acrylic acid)	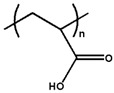	4.75
	Poly(methacrylic acid)	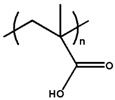	6.30
	Poly-sulfonamides	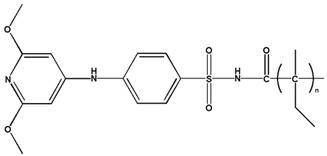	6.80

**Table 3:** Acid-labile chemical bonds and their degradation products.

	Acid-Labile Bond		Chemical Structure	Degradation Products	pH Range	Ref.
1	C=N bond	Hydrazone	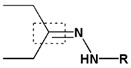	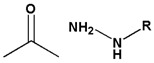	~5.0	[124,125]
		Imine	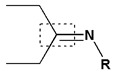	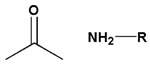	~6.8	[126,127]
		Oxime	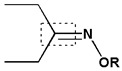	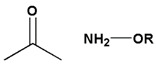	~5.0	[128,129]
2	Acetal and Ketal bond		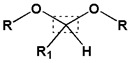	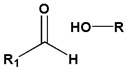	5.0~5.4	[130,131,132]
			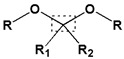	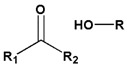		
3	Amide bond	Beta- carboxyl amide	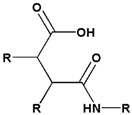	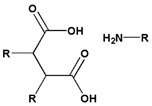	4.5~6.0	[133,134]
		*Cis*- aconityl amide	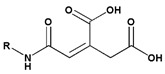	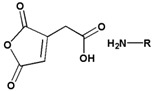		
4	Ester bond	Ester	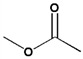	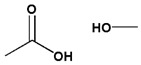	~6.0	[135,136]
		Succinic ester	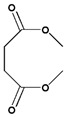	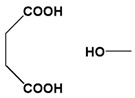		
		β-Thiopropionate